# Dengue fever in renal transplant patients: a systematic review of literature

**DOI:** 10.1186/s12882-016-0428-y

**Published:** 2017-01-13

**Authors:** Ranga Migara Weerakkody, Jean Ansbel Patrick, Mohammed Hussain Rezvi Sheriff

**Affiliations:** 1 University Medical Unit, National Hospital of Sri Lanka, 79, Regent Street, Colombo 9, Sri Lanka; 2 Addenbrookes Hospital, Cambridge University Hospitals, Hills Road, Cambridge, CB2 0QQ UK; 3 Department of Clinical Medicine, Faculty of Medical Sciences, Sir John KotelawalaDefence University, Ratmalana, Sri Lanka

**Keywords:** Dengue, Renal transplant, Immunosuppression, Graft failure

## Abstract

**Background:**

Dengue fever in renal transplanted patients has not been studied well, and we review all the literature about episodes dengue fever in renal transplant patients.

**Methods:**

The aim was to describe clinico-pathological characteristics, immunosuppressive protocols, need renal outcome and mortality. PubMed, LILACS, Google Scholar and Research Gate were searched for “Dengue” and “Renal/Kidney Transplantation” with no date limits. Hits were analyzed by two researchers separately.

**Results:**

Fever, myalgia, arthralgia and headache was significantly lower than normal population, while pleural effusions and ascites were observed more. Incidence of severe dengue is significantly higher among transplant patients in this review, as well as they had a significantly higher mortality (8.9% vs 3.7%, *p* = 0.031). Age, period after transplantation and immunosuppressive profile had no effect on disease severity, mortality or graft out come. Presence of new bleeding complications and ascites was associated with more severe disease (*p* < 0.001 and *p* = 0.005), death (*p* = 0.033) or graft loss (*p* = 0.035). Use of tacrolimus was associated with new bleeding complications (*p* = 0.027), and with ascites (*p* = 0.021), but not with thrombocytopenia. 25% of patients with primary disease fail to mount an IgG response by 15 weeks of the illness. 58.9% had graft dysfunction during illness. Postoperative transplanted patients were at risk of severe disease and unfavorable outcome.

**Conclusions:**

The physical and laboratory findings in dengue fever in renal transplanted patients differ from the general population. Some degree of graft dysfunction is common during the illness, but only a minority develops graft failure.

## Background

Dengue is the most rapidly spreading mosquito-borne viral disease in the world. In the last 50 years, incidence has increased 30-fold with spread to new countries, and from urban to rural settings. An estimated 50 million dengue infections occur annually and approximately 2.5 billion people live in dengue endemic countries [1]. The illness ranges from being asymptomatic or a mild flu like illness (dengue fever, DF) associated with a rash, to a more severe form with plasma leakage, hemorrhage (dengue hemorrhagic fever – DHF), shock and multi organ failure (dengue shock syndrome-DSS). Liver failure, cardiac, neurological or hematological complications may occur asatypical complications, termed as extended dengue syndrome [2–5].

With the advancement of immunosuppression, renal transplant has become the management of choice for end stage renal disease (ESRD). Some transplant recipients are living in hyper-endemic and endemic areas for dengue, and are at risk of developing the disease. The affected countries are places of great tourist attraction, and travelling transplant recipients are at risk too. Dengue is described as a mild disease in renal transplant patients [6], but severe morbidity (graft failure needing dialysis and graft nephrectomy) [7], and mortality [4], had been reported. This paper reviews demographical, clinic-pathological and immunosuppression profiles of the renal transplant patients with confirmed dengue infection.

## Methods

We searched PubMed, LILACS, Google Scholar and ResearchGate for publications by key words / MeSH terms “dengue” AND (“renal transplant” OR “kidney transplant”) in title, abstract or full text, with no date limits. The search was performed in December, 2015. The two investigators (RMW and JAP) independently gone through the abstracts to identify relevant papers. We selected all the papers that had information on patients with renal transplant who suffered from dengue infection. There were no randomized trials; all the papers were cross sectional studies or case series / reports. Due to the rarity of the condition, we included all of them in this review. We also searched related publications and papers referenced in review articles. Full papers of selected studies were read through by the two investigators, who extracted relevant data. Duplicate publications of the same data were excluded. The results of the larger studies are presented as it is, and findings of all the case reports and the case series (composite group) were summarized and analyzed. Data of two patients from an unpublished work from RMW was added to the review. Post operative period was defined as first 14 days after the transplant. Whenever possible the combined statistics across all the cross sectional studies / case reports was calculated. SPSS 16.0 was used for the analysis. For summary data the Fisher’s exact value calculator from the web site GraphPad Software® was used [8], while for calculating pooled averages and standard deviations, tools from University of Baltimore web site [9] was utilized. Forest plots were created using RevMan 5.3® from Cochrane Reviews [10]. Mann Whitney *U* test and Fishers exact tests was used to compare groups as normality assumption was violated by the continuous variables, and of small values in cross-tabs. Risk of bias analysis was performed by two the authors independently, using ROBINS-I tool. Inter-rater agreement was 100%.

## Results

**Fig. 1:**
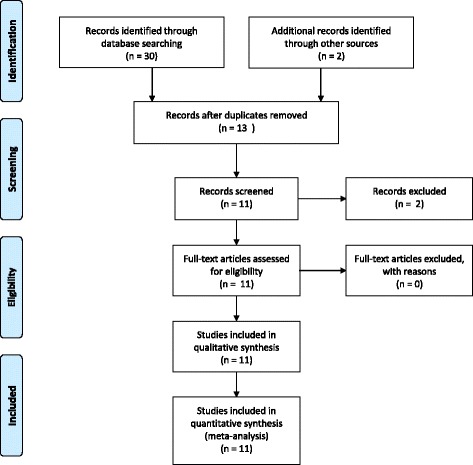
PRISMA flow diagram showing study identification, screening, eligibility and inclusion

The search of PubMed, Google Scholar, LILACS and ResearchGate resulted in 33 hits. Once the duplicates were removed we were left with 13 records. Three records were responses to previous case reports, and hence were excluded (See Fig. 1). We reviewed two large cross sectional studies, three case series and four case reports. In one study only the abstract was available [11], and in the other immunosuppression protocol was absent [12], however we used data on symptomatology, lab investigations and mortality. The total number of subjects described in literature was 168. Table 1 gives a summary of the reviewed studies. Most of the studies had information on basic demographics (age and gender), time since transplant, details of immunosuppression, symptomatology, basic laboratory results, severity of disease, complications, graft failure, length of hospital stay, method of confirmatory diagnosis and eventual outcome.

Nasim et al. [6] has done the largest study on DF in RT involving 102 subjects (Male 73.7%) in city of Karachchi for a period of 2 years. The conclusions of their study are summarized in Table 2. Mean increase of creatinine was 66 ± 48 umol/L. Azavedo et al. [2]. Reported a cross sectional of 27 (Male 67%) patients from Brazil, and found that, the symptoms are similar to that of non-transplant population other than for arthralgia. The outcome of transplanted patients was similarto the normal dengue population. Immunosuppression did not affect the outcome. Mean increase of creatinine was 35.7%.

**Table 1:** The studies selected for review and their basic characteristics

Study	Country of origin	Number of cases	Type of study
Azavedo et al. [2]	Brazil	27	Cross sectional
Costa et al. [7]	Brazil	10	Case series
Maia et al. [12]	Brazil	2	Case series
Mayor et al. [11]	Paraguay	8	Cross sectional
Nasim et al. [6]	Pakistan	102	Cross sectional
Park et al. [3]	South Korea	1	Case report
Prasad et al. [4]	India	8	Case series
Raynaud et al. [13]	Singapore	6	Case series
Tan et al. [5]	Singapore	1	Case report
Tangnararatchak [14]	Thailand	1	Case report
Weerakkody	Sri Lanka	2	Unpublished data

**Table 2:** Conclusions of the study performed by Nasim et al

•	DHF / DSS commoner in subjects on high dose steroids
**•**	Secondary infection on cyclosporine, a significantly lesser proportion of patients presented, with less severe disease DHF/DSS vs DF (*p* = 0.04)
**•**	Fever is commoner in patients taking low dose steroids to patients on high-dose steroids (*p* = 0.013)
**•**	The anti-mitotic agents (azathioprine (AZA) or mychophenolate mofetil (MMF)) have no effect; on the severity or duration of thrombocytopenia;leucopenia; and occurrence of gastro-intestinal symptoms.
**•**	Mean duration of thrombocytopenia is longer in patients on regimens containing tacrolimus
**•**	Patients on tacrolimus containing regimens have a higher mortality (*p* = 0.02)
**•**	Percentage rise in creatinine from pre-dengue levels was higher in DHF/DSS patients than in DF patients (*p* < 0.001)
**•**	Majority (85.7%) who had graft dysfunction, creatinine returned to base line by 12.6 days, whereas in 14.3% it persisted beyond 6 weeks.
**•**	All the patients who died had graft dysfunction
**•**	Of 21 patients who were IgM positive and IgG negative in the initial sample, 10 (48%) had not mounted an IgG response by an average of 15 weeks
**•**	No statistically significant difference was found in the number of years as transplantation of those who survived vs. those who died

### Composite group outcomes

This group consisted of 29 patients (Male 48.3%), from six countries, across six studies [3–5, 7, 13, 14]. As explained earlier in the text, two studies were left out in forming the composite group. The mean age was 40.2 ± 14.4 years, and median duration from transplant was 42 months (mean 51.3 ± 55.3 months). Table 3 summarizes the immunosuppressive profile. Symptom and Signs analysis show fever on presentation (91.3%), myalgia (95.5%), arthralgia (25%), new bleeding complications (34.8%), headache (91.3%), diarrhea (15.3%), pleural effusions (84.6%) and ascites (38.5%). Laboratory test wise, thrombocytopenia (87.0%), leucopenia (51.7%), liver function test abnormalities (69.0%) and graft dysfunction (37.9%). severe dengue infection (DHF / DSS) accounted for 34.5%. The diagnosis confirmation was done serologically in 44.8 and 55.1% using polymerase chain reaction or dengue NS1 antigen. Out of the 29 patients 3 (10.8%) succumbed to illness and 2 (6.9%) lost their grafts.

**Table 3:** Immunosuppressive profile of the composite group

Medication	Number	Percent
Azathioprine	3	10.3
Cyclosporine	15	51.7
MMF	23	79.3
Steroids	27	93.1
Tacrolimus	13	44.8

**Table 4:**
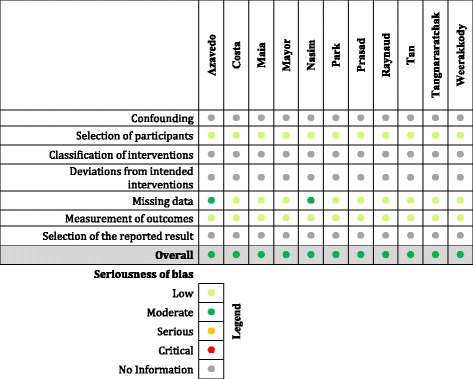
Risk of bias summary for the studies. The colored bullet signifies the risk of bias for each study, under sections described in ROBINS I checklist. Light green - low, dark green - moderate, orange - serious, red - critical, ash - risk of bias cannot be assessed due to lack of information.

We have analyzed the risk factors for signs and symptoms as well as mortality in the composite group. Out of the large number of comparisons only the outcomes described in Nasim et al. and Azavedo et al. was analyzed. Gender and immunosuppressive profile was not associated with disease severity or the outcome of the disease. Presence of new bleeding complications and ascites was associated with more severe disease (Fishers’ Exact, *p* < 0.001 and *p* = 0.005) as well as with adverse outcome of death or graft loss (Fishers’ Exact, *p* = 0.033 and *p* = 0.035). Use of tacrolimus was associated with new bleeding complications (Fishers exact test, *p* = 0.027), and with ascites (Fishers exact test, *p* = 0.021), but not with thrombocytopenia. We could not run a multiple logistic regression with new bleeding manifestations, severity of disease, use of tacrolimus and presence of ascites as covariates, due to small number of records (*n* = 13).

### Synthesis of results

**Fig. 2:**
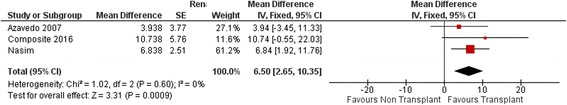
Forest plot of mortality statistics among three study groups

In the synthesis of the results, we tried to pool the common parameters to achieve single values of central tendency and proportion. The combined results included 168 patients (Male 67.9%) with a mean age of 38.9 ± 13.8y (*n* = 66), and a mean time since transplant 53.6 ± 60.8 months (*n* = 66). The duration of transplant did not have effect on the presentation or the outcome. Mean increase of creatinine was 67.1% (*n* = 131) from the baseline. Table 5 summarizes the combined results across all the studies. We found new bleeding complications or diarrhea to show no difference in frequency compared to the normal population [15]. Fever, myalgia, arthralgia and headache were significantly lower than normal population, while pleural effusions and ascites were observed in a greater frequency. Incidence of severe dengue is significantly higher among transplant patients in this review, as well as they had a significantly higher mortality. The mortality was significantly higher than that of severe dengue fever (8.9% vs 3.7%, t = 2.27, *p* = 0.031). The forest plot for the mortality results are shown in Fig. 2.

**Table 5:** Summary of immunosuppressive profile, symptoms and signs, laboratory findings, severity of disease, confirmation of diagnosis and outcome, and comparison with general population

	*n*	%	Prevalence7^a^ (*n* = 81,327) %	*p*
Immunosuppression				
Azathioprine	166	51.8		
Cyclosporine	166	27.1		
MMF	166	43.4		
Steroid	166	98.8		
Tacrolimus	166	25.9		
Symptoms and Signs				
Fever at presentation	162	86.4	98.8	0.006
Myalgia	159	47.0	92.4	<0.001
Arthlagia	39	20.2	76.4	<0.001
New bleeding complications	127	14.9	10.2	0.147
Headache	133	34.6	95.8	<0.001
Diarrhea	13	15.3		0.122
Pleural effusions	115	17.4	2.2	<0.001
Ascites	23	34.8	1.1	0.003
Laboratory findings				
Thrombocytopenia	127	93.7		
Leucopenia	133	66.9		
LFT abnormalities	39	71.8		
Graft dysfunction	141	58.9		
Severity of disease				
DHF / DSS	168	16.0	3.7	<0.001
Confirmation of disease				
IgM	168	87.5		
RT PCR / NS1	168	13.7		
Outcome				
Alive with a functioning graft	168	84.5		
Graft failure	168	6.5		
Death	168	8.9	0.062 ^b^	<0.001

*Abbreviations*: *LFT* liver function tests, *RT PCR* reverse transcriptase polymerase chain reaction, *NS1* non specific antigen 1

^a^Prevalence of non-transplant population given by Casali CG et al. [14]

^b^Frequency of DHF given in Casali et al. is 3.7%. The mortality of DHF is 3.21% while that of DF is 0.026%. The combined mortality calculated as 0.062%

In the study by Nasim et al., patients who are on tacrolimus had a higher mortality (*n* = 102, *p* = 0.031, Fishers’ Exact), but in the final analysis showed no association (*n* = 131, *v*0.28, Fishers’ Exact). Being in the post operative period, increased the risk of dengue hemorrhagic fever (*n* = 31, *p* = 0.001, Fisher’s Exact) as well as, ending up dead or with a failed graft (*n* = 31, *p* = 0.044, Fisher’s Exact).

### Risk of bias across studies

All of these studies are either case reports or cross sectional surveys, and as a result have selection bias. Patients who have severe disease are more likely to turn up at their physicians’ office rather than a person having a mild flu like illness. Hence all the studies are biased towards severe manifestations of the disease, as a result higher death rates and morbidity. A formal risk of bias analysis was performed using ROBINS-I tool, and the results are described in Table 4. Publication bias is obvious, due to the rarity of the clinical circumstances.

## Discussion

Renal transplanted patients with Dengue Fever exhibit different clinical features to that of general population. Fever, headache, myalgia and arthralgia are less common in transplant population, while incidence of ascites and pleural effusions is higher. Transplanted patients had a higher incidence of severe dengue fever and a higher mortality. Nasim et al. report a significantly lower rate of severe primary dengue infection, in patients taking low dose steroids. Additionally, they report a lower mortality from secondary infection, in patients whom on cyclosporine based regimens. We suggest that deciding whether a dengue infection is primary or secondary should be done in care in transplant population. Nasim et al. reports that 10 out of 21 of their patients who were IgG negative and IgM positive −22.7% of patients diagnosed of primary infection-failed to mount an IgG response after 15 weeks of the illness. This raises the validity of the diagnosis of primary dengue infection in transplanted population in previous studies. We suggest that the distinction between primary and secondary infections in transplanted patients should be disregarded, given the unreliability of the antibody response following the infection. The higher mortality associated with tacrolimus [6] was no longer significant when composite group was added to the analysis. Serological diagnosis is still the most popular form of diagnosis, but RT-PCR and NS1 antigen which provide speedy confirmatory diagnosis are gaining popularity. Nearly sixty percent developed graft dysfunction, with a mean creatinine rise of 61.7%, during the course of illness and from most of them recovered. Mortality was 8.9% and graft loss was about 6.5%, and is not due to direct effects of dengue. Mortality is way higher than for the normal population (0.062%) [1], but as discussed above, the samples were subjected to heavy selection bias as only the patients with most severe symptoms would have undergone confirmation of the dengue infection, while others would have been treated as for a non-specific viral illness. The results show post operative patients being more vulnerable to severe disease as well as reaching the end point of death or losing the graft.

## Limitations

Most of the studies used to in this review are cross sectional studies and case series / reports. Hence the quality of evidence is not as high as that of a randomized trial. The selection bias as described earlier makes the generalization of the results difficult to the whole population. Since ROBINS—I was developed mainly for case control and cohort studies, we cannot get and accurate estimate of bias as well. Still the evidence is helpful in the management of inward patients with dengue fever.

## Conclusions

The physical and laboratory findings in dengue fever in renal transplanted patients do not differ from the general population. Early post-operative patients are vulnerable to the disease more, with severe form of disease and higher complication rates.

## Abbreviations

AZA: Azathioprine
DF: Dengue fever
DHF: Dengue hemorrhagic fever
DSS: Dengue shock syndrome
ESRD: End stage renal disease
LFT: Liver function tests
MMF: Mychophenolate mofetil
NS1: Non specific antigen 1
RT: Renal transplant
RT-PCR: Reverse transcriptase-polymerase chain reaction
